# Supplementary material for: Are carbon emissions trading and green financial instruments synergistic? -Comprehensive quantitative research based on content analysis
Source: PLoS One. 2024 Mar 7;19(3):e0298601. doi: 10.1371/journal.pone.0298601 (PMC10919601; doi:10.1371/journal.pone.0298601)
Supplement: S1 Data — (ZIP) [file pone.0298601.s001.zip › Data/地级市Z_F工作报告.zip!/║╙▒▒/║¬╡a╩╨/└┤╫╘╣1⁄2╓┌║┼í╛┬φ┐╦╩2╛▌═°í┐_2016.docx]

各位代表：

现在，我代表市人民政府向大会作工作报告，请各位代表连同《邯郸市国民经济和社会发展第十三个五年规划纲要（草案）》一并审议，并请市政协委员和列席会议的同志提出意见。

一、“十二五”时期经济社会发展回顾

“十二五”时期，是邯郸发展历程中极不平凡的五年。面对复杂严峻的经济形势，市政府在省委、省政府和中共邯郸市委的坚强领导下，在市人大、市政协的监督支持下，深入学习贯彻习近平总书记系列重要讲话精神，主动适应经济发展新常态，紧紧围绕建设宜居宜业宜游富强邯郸、美丽邯郸的战略目标，攻坚克难，砥砺奋进，较好完成了“十二五”规划主要目标任务，在全面建成小康社会进程中迈出坚实步伐。

过去五年，是经济平稳较快发展、综合实力显著增强的五年。着力上项目、稳增长，持续开展项目建设“三集中”活动，累计建设省重点项目194个、市重点项目356个，美的工业园、新兴特种管材、银隆新能源汽车、五粮液灌装基地等一批重大产业项目建成投产。全市生产总值由2010年的2361.6亿元增加到2015年的3145.4亿元、年均增长8.6%，全部财政收入由244.2亿元增加到307.7亿元、年均增长4.7%，一般公共预算收入由115.9亿元增加到190.6亿元、年均增长10.5%。规模以上工业增加值达1107.2亿元、年均增长9.4%，固定资产投资3443.3亿元、年均增长18.1%，社会消费品零售总额1358.6亿元、年均增长13.6%。全市经济运行在逆势中调速不减势、量增质更优，为今后更好更快发展打下了坚实基础。

过去五年，是产业结构持续优化、发展方式加快转变的五年。三次产业结构由2010年的13.0: 54.2: 32.8，调整优化为12.8：47.7：39.5。传统产业改造提升步伐加快，“三年千项”技改工程圆满完成，技改投资年均增长20.5%。累计压减炼铁产能795万吨、炼钢产能542万吨、焦炭产能313万吨、水泥产能1260万吨，六大高耗能行业增加值占规模以上工业比重比2010年下降15.4个百分点，预计单位生产总值能耗累计下降20%以上，提前一年完成“十二五”节能目标，成为国家循环经济试点示范市。战略性新兴产业规模不断壮大，装备制造业、高新技术产业增加值占规模以上工业比重分别达13.1%和10.8%，比2010年提高7.4个和7.7个百分点，装备制造业成为我市第二大支柱产业。现代服务业加速发展，服务业增加值占生产总值比重比2010年提高6.7个百分点，旅游业总收入年均增长34.8%，国家4A级以上景区达14家。现代农业稳步发展，2015年粮食总产达542.5万吨，比2010年增加66.4万吨，建成华北首个“吨粮市”，连续三年荣获全国粮食生产先进市，曲周农业科技园区升级为国家级园区，建成省级农业科技园区11家，省级以上重点龙头企业达62家，农业产业化经营率达66.2%，比2010年提高6.4个百分点，土地流转率达27%。科技创新能力增强，国家高新技术企业达159家，科技型中小企业达4392家，专利授权7322件，荣获省级以上科技进步奖95项。

过去五年，是新型城镇化战略深入实施、城乡面貌大幅提升的五年。城镇规划体系日趋完善，部分县区行政区划调整稳步实施。东区建设全面启动，新建道路11条，东郊热电、客运枢纽、科技中心、青少年活动中心等一批工程开工在建。老城区改造深入推进，文化艺术中心、4座立交桥建成投用，滏阳河改造提升工程一期通航，新改建城市道路84条、小街巷278条，改造完成城中村和旧城区片59个，市政设施不断完善，园林绿化水平进一步提高。我市列入国家智慧城市试点，城市精细化管理水平得到提升。重大基础设施保障能力显著增强，京广高铁、邯黄铁路、邯长邯济铁路扩能改造、邯大高速公路、京港澳高速改扩建、机场二期等交通工程竣工投用，南水北调中线工程建成通水，总干渠沿线地区用上了长江水。县城建设扩容升级，建成6个国家级园林县城、8个省级园林县城，实现县县通天然气。19个镇列入全国重点镇，建设美丽乡村972个。全市常住人口城镇化率达51.38%，比2010年提高7个百分点。

过去五年，是体制机制革故鼎新、改革开放全面深化的五年。政府职能加快转变，坚持简政放权、放管结合、优化服务，市级审批事项由2010年的206项精简到152项，“六个清单”“三级平台”制度全面建立，市县乡政府机构改革全面完成。商事制度改革成效明显，实施“三证合一”“一照一码一证一章”，大众创业、万众创新氛围日益浓厚，全市市场主体达38.1万户，是2010年的3.7倍。农村土地确权登记颁证面积完成60.8%，96家市县属国有企业实施改制破产重组，工商、质监和食品药品监管体制改革深入推进，供销社改革成效明显。抢抓京津冀协同发展战略机遇，累计与央企签订合作协议87个，北大邯郸创新研究院挂牌成立，教育、医疗、旅游等多领域合作不断深化。荷兰哈克、德国优布劳、白俄罗斯戈梅利等一批世界知名企业落户邯郸，实际利用外资累计达40.9亿美元、年均增长10.1%，出口总值累计达76.5亿美元、年均增长14.6%。金融创新力度加大，挂牌上市企业达56家，驻邯银行业金融机构达20家，邯郸银行跨入世界千强银行。邯郸经济开发区晋升为国家级经济技术开发区，冀南新区成为省级高新技术产业开发区，冀津循环经济产业示范区获批成立，全市省级经济开发区达20家。民营经济增加值达2051.3亿元，年均增长10%。

过去五年，是生态环境有效改善、可持续发展能力提高的五年。坚决向大气污染宣战，大力实施减煤、控车、降尘、治企等重点工程，通过“煤改气”“煤改电”“散改型”实施清洁能源替代，预计煤炭消费量比2012年削减570万吨。完成钢铁、水泥、电力、焦化、玻璃五大行业359项污染治理工程，淘汰黄标车11.5万辆，拆除烟囱2872根，淘汰改造燃煤锅炉2320台，关停取缔“三小”企业4629家，累计实施减排工程2480项，如期完成“十二五”减排目标。2015年空气质量综合指数、PM2.5平均浓度分别比2013 年下降33.9%和34.1%。加强山水林田湖整体修复，治理水土流失面积750 平方公里，13个县列入地下水超采治理国家试点，形成地下水压采能力2.75亿立方米，我市列入全国水生态文明城市建设试点。“绿美邯郸”建设成效明显，累计造林绿化190万亩，全市森林覆盖率达27.7%，比2010年提高7.5个百分点，荣获全国绿化模范城市。

过去五年，是社会事业全面进步、人民群众得到更多实惠的五年。每年办理一批民生实事，全市财政用于民生支出累计达1279.7亿元，比“十一五”提高136%。城镇、农村居民人均可支配收入分别达到24630元和11247元，比2010年增长61.3%和89.6%。累计投入各类扶贫资金39.3亿元，58.4万贫困人口实现稳定脱贫。城镇累计新增就业59.7万人，转移农村富余劳动力35.8万人。社保体系日益完善，城乡低保标准分别比2010年提高83%和94%，新农合参合率达98.69%，城乡居民基本养老保险、大病保险实现全覆盖，城镇企业退休人员基本养老金连续11年上调，农村互助幸福院、城市社区居家养老服务中心覆盖率分别达82%和65%。开工建设保障性住房6万套，改造农村危房4.6万套，解决了275.9万农村人口饮水安全问题。各级各类教育长足发展，教育教学质量进一步提高，市一中、二中新校区建成投用，办学条件明显改善。公共文化服务体系不断完善，中国吹歌节、中原民间艺术节等文化活动丰富多彩，国家级非物质文化遗产达27项，全国重点文物保护单位达34处，赵王城遗址列入国家考古遗址公园立项名单。医疗卫生和计划生育服务体系日益健全，县级公立医院综合改革实现全覆盖，城市公立医院改革全面启动。全民健身运动广泛开展，成功举办第十二届国际太极拳运动大会。平安邯郸建设深入推进，安全生产形势总体稳定，食品药品安全监管力度加大，依法打击和处置非法集资，构建“三网合一”社会治安防控体系，刑事立案总量比2010年下降57.2%，“两抢一盗”案件下降21%，人民群众的幸福感和满意度明显提升。文明城市创建扎实推进，荣获全国未成年人思想道德建设工作先进城市。国防动员和双拥共建深入开展，荣获全国双拥模范城“七连冠”。《邯郸市志》一二卷出版发行。民族宗教、广播电视、新闻出版、外事侨务、邮政通信、人民防空、防震减灾、妇女儿童、援疆援藏、扶残助残、档案、老龄、气象等事业都取得了新成绩。

同时，我们不断加强政府自身建设，自觉接受人大、政协监督，人大代表建议、政协委员提案按时办复率均达100%，切实加大审计监督、行政监察和反腐败工作力度，服务政府、法治政府、廉洁政府建设迈出新步伐。

刚刚过去的2015年，是“十二五”收官之年。我们扎实开展“三严三实”专题教育和解放思想大讨论，统筹做好稳增长、调结构、促改革、治污染、惠民生等各项工作，全市经济社会发展稳中有进、稳中有新、稳中向好。

一是经济运行企稳回升。积极应对经济下行压力，研究出台了“房九条”“抵质押九条”“科技九条”“金十条”等一系列创新性政策措施，市级领导带头联系66家重点企业，实施金桥帮扶行动，促进了经济平稳增长。主城区住宅销售面积同比增长51.1%，新认定科技型中小企业2476家，全市新增贷款438.9亿元，存贷比达67.73%，创历史新高。全市生产总值增长6.8%，一般公共预算收入可比增长6.5%，规模以上工业增加值增长4.7%，固定资产投资增长11.4%，社会消费品零售总额增长9.4%，出口总值增长5.5%，城镇、农村居民人均可支配收入分别增长8.5%和8.7%。

二是项目建设势头强劲。省、市重点项目分别完成投资252.5亿元和965亿元，超年计划31.7个和20.6个百分点。市级成立了4个招商部，市县主要领导带头招商，集中签约项目151个，超百亿、超五十亿元项目分别达5个和25个，超额完成“215”目标。与中国恒天、中国建材、中化集团等21家央企签订31个合作协议，邯郸华耀城、华强中华成语文化博览园、中材风电叶片等一批大项目、好项目成功落地。

三是结构调整力度加大。工业转型步伐加快，永洋特钢退城搬迁升级改造项目开工建设，太行和冀南钢铁退城进园升级改造、新峰煤制天然气等项目有序推进，装备制造、高新技术、现代物流三大新兴产业投资增长13.7%，高于全市固定资产投资增速2.3个百分点。现代服务业发展提速，邯郸电商谷、昊远跨境电商产业园等一批电子商务项目开工建设，邯郸海关挂牌开关，国际陆港开港运营，娲皇宫晋升为5A级景区，广府古城通过5A级景区景观质量评审。现代农业加快发展，建成3个省级和7个市级现代农业园区。

四是改革创新活力增强。非行政许可审批事项全部取消，市民车、市民网、市民卡赢得群众普遍欢迎。新增省级企业技术中心7家、省级工程技术研究中心5家、中国驰名商标3件。组建了市金融资本服务中心、市科技金融服务中心、市股权交易服务公司，邯郸农商银行挂牌成立，设立2家科技支行。大学生创业园、众创空间等创业平台蓬勃发展。

五是空气质量得到改善。总投资10.2亿元，对主城区及周边外扩5公里区域内275个村庄（社区）实施了“煤改气”工程，10万余户居民用上天然气，2015年12月份主城区二氧化硫同比下降38.9%。48台燃煤发电机组完成超低排放改造，486座实心粘土砖瓦窑和1426处非法加油站点全部关停取缔。拆除烟囱1222根，淘汰改造燃煤锅炉1365台。加强扬尘和机动车污染治理，市区北环路实行载重货车绕行。空气质量优良天数150天，同比增加62天；重度及以上污染天数33天，同比减少58天。

六是城乡建设扎实推进。东区供排水、供气、供热等市政设施开工建设，东军师堡完成征迁，西军师堡回迁房建设加快，“三路一场”回迁房竣工入住，河北工程大学迁建启动。滏阳河酒吧街基本建成，串城街改造加紧施工，世纪大街、东柳大街南延竣工通车，“两高”沿线和北环路综合整治成效明显，绕城高速公路实现闭合通车。县城承载力明显增强，按照“3+X”思路，建成40个美丽乡村精品村，馆陶县寿东村被评为全国十大最美乡村。

七是社会事业亮点纷呈。均衡教育、医疗卫生等11个方面75件民生实事如期完成。我市被评为全国电子社保示范城市。中考、高考成绩实现历史性突破，18名学子被清华和北大录取。电视剧《太行山上》在央视热播，并荣获第三十届“飞天奖”优秀电视剧奖，中国成语大会总决赛在我市举行，中国海军“邯郸舰”入列服役，第十二届市运动会成功举办。

在看到成绩的同时，我们也清醒地认识到，邯郸正处在滚石上山、爬坡过坎的关键时期，发展中还存在不少困难和问题。一是经济下行压力依然较大，企业生产经营困难，稳增长的任务十分艰巨；二是产业结构偏重，重工业占比仍达七成以上，新兴产业还没有挑起大梁，科技创新能力不强，新旧动能转换不快，发展的质量和效益需进一步提高；三是资源环境约束日益凸显，化剩治污任务繁重，摘掉大气污染“黑帽子”还需付出艰苦努力；四是财政收支矛盾加剧，收入质量不高，刚性支出不断增多，事关群众切身利益的民生问题解决得还不够好；五是发展环境仍需优化，一些地方和部门行政效能还不高，服务意识还不强，不同程度存在不作为、慢作为、乱作为问题。对此，我们一定高度重视，深入研究，逐一破解。

各位代表！回顾“十二五”改革发展历程，我们遇到的挑战前所未有，取得的成绩来之不易。这是省委、省政府和中共邯郸市委正确领导的结果，是市人大、市政协监督支持的结果，是全市广大干部群众团结奋斗、拼搏实干的结果。在此，我代表市人民政府，向全市人民，向人大代表、政协委员，向各民主党派、工商联、无党派人士和人民团体，向驻邯人民解放军、武警官兵和政法干警，向中直、省直驻邯各单位，向各位老领导，向所有关心支持邯郸发展的各界人士，表示崇高的敬意和衷心的感谢！

二、“十三五”时期主要目标和任务

“十三五”时期，是全面建成小康社会的决胜阶段，也是决定邯郸发展前途命运的关键五年。在前进的道路上，挑战多但机遇更多，困难大但优势更大，我们对未来充满信心。

“十三五”时期，是我市发展历史上重大机遇最为集中的时期。我国经济长期向好的基本面没有改变，发展仍处于大有作为的重要战略机遇期。京津冀协同发展、“一带一路”、中原经济区和环渤海地区合作发展等国家战略的深入实施，为我们在更大范围、更广领域、更高层次聚集先进生产要素，推进更高水平的双向开放，提供了广阔空间。

“十三五”时期，是加快发展转型突破最为宝贵的时期。中央大力推进供给侧结构性改革，将出台一系列重大利好政策，为我们转型发展营造了良好的政策环境；《中国制造2025》、“互联网+”行动计划等战略的深入推进，为加快产业结构调整创造了有利条件；省委、省政府对邯郸发展寄予厚望，要求在深化改革、转型升级中闯出一片新天地，为我们指明了发展方向。只要我们抓住机遇，乘势而上，就一定能够迈入结构优、质量高、效益好的发展轨道。

“十三五”时期，是各种优势潜力最能有效释放的时期。我市具有独特的省际区位、产业基础、投资潜力等组合优势，经过近几年稳步较快发展，“硬地起跳”的基础更加牢固。我市连续四年实行“三集中”工作法，建设了一批大项目好项目，积聚了强大的发展势能，必将对经济增长带来强劲的拉动力。

“十三五”时期，是破解难题补齐短板最为紧要的时期。对照全面小康标准，我市全面建成小康社会的实现程度还不高，特别是区域协调发展、经济转型升级、科技创新能力、新型城镇化水平等方面还有一定差距。补齐短板是“十三五”时期的重要任务，我们一定要找准薄弱环节，破解发展难题，构建全面、平衡、协调发展的新格局，增强综合实力和竞争力，提升全市人民的幸福指数。

“十三五”时期我市经济社会发展的指导思想是：全面贯彻党的十八大和十八届三中、四中、五中全会精神，以邓小平理论、“三个代表”重要思想、科学发展观为指导，深入贯彻习近平总书记系列重要讲话精神，按照“五位一体”总体布局和“四个全面”战略布局，贯彻落实创新、协调、绿色、开放、共享的发展理念，认真落实省委八届十二次全会和市委八届七次全会部署，扭住发展第一要务不放松，以提高发展质量和效益为中心，突出加快发展、转型突破、补齐短板、做美生态四大战略任务，坚守发展、生态、民生三条底线，着力在供给侧结构性改革上取得突破，重点推进新型工业化、信息化、城镇化和农业现代化，打造京津冀联动中原的区域中心城市，坚决打赢脱贫攻坚战，确保如期全面建成小康社会，把宜居宜业宜游富强邯郸、美丽邯郸的建设事业奋力推向新阶段。

统筹考虑贯彻五大发展理念和我市经济社会发展需要，“十三五”时期主要目标设定为28项，其中约束性指标15项，预期性指标13项。总体目标概括为“四超”和“一个全面建成”，即在提高发展平衡性、包容性、可持续性的基础上，主要经济指标增速超过全省平均水平，生产总值年均增长8%左右；城乡居民人均可支配收入超过全省平均水平，年均增长9%；常住人口城镇化率、户籍人口城镇化率超过全省平均水平，分别达到60%以上和45%以上；主要污染物排放削减率超过全省平均水平，环境治理取得明显成效；确保到2020年如期全面建成小康社会。

根据上述目标要求，围绕两大发展定位（城市定位：京津冀联动中原的区域中心城市、环渤海辐射中西部的重要门户城市；产业定位：“五基地一中心”），今后五年，重点抓好以下六大任务。

（一）坚持走创新发展之路，加快实现经济增长动能转换。把创新作为加快发展的第一动力，让创新活力竞相迸发，以创新力提升竞争力。以科技创新为引领。实施五大创新工程，创建一批工程技术研究中心、企业技术中心、重点实验室，实施一批重大科技项目，引进培养一批高层次科研人才，培育壮大高新技术企业，推动科技型中小企业裂变式增长。以改革创新求突破。深化行政管理体制改革，大力简政放权，提高行政效能。健全现代产权制度，推进国有企业分类改革。创新政府投资方式，推广政府与社会资本合作（PPP）模式。增多做优金融主体，激活做大金融市场。深化农村集体产权制度改革，统筹推进社会领域各项改革。以大众创新激活力。开展“双创”邯郸行动，完善全链条服务体系，搭建一批新型创业平台，打造创客“栖息地”和“聚集区”，在全市形成大众创业、万众创新的新局面。

（二）坚持走转型发展之路，全面推进产业向中高端迈进。把转型作为加快发展的核心任务，从供给侧和需求侧两端发力，做好加减乘除法，培育新的经济增长动力。强化产业支撑。重点打造精品钢材、装备制造、食品加工、节能环保、新能源汽车和现代物流六大千亿元产业，建设“五基地一中心”。调整优化结构。引导传统产业转型发展，加大化解过剩产能力度，确保到2017年，完成压减炼铁产能1614万吨、炼钢产能1204万吨任务，规模以上重工业占GDP比重下降到25%以下。培育壮大战略性新兴产业，使之尽快成为经济发展的顶梁柱。加快发展总部经济、创意设计、电子商务等生产性服务业，培育壮大文化旅游、商贸服务、健康养老等生活性服务业，服务业增加值占全市生产总值比重提高到45%以上。夯实发展基础。在提高粮食综合生产能力的基础上，加大农业结构调整力度，推进现代农业园区和优质农产品基地建设，发展高效、优质、生态、品牌、安全农业，建设现代农业强市。

（三）坚持走协调发展之路，着力形成城乡区域平衡发展结构。把协调作为加快发展的内在要求，不断补齐发展短板，拓展发展空间。推进城乡一体发展。坚定不移走新型城镇化道路，把海绵城市、智慧城市理念贯穿始终，有序推进行政区划调整，加快东区开发建设，完善老城区配套设施，着力打造区域性中心城市。实施县城建设三年攻坚行动，做大县城建成区规模，支持有条件的县撤县建市、有规模的县建成中等城市，建设一批特色小城镇，基本实现美丽乡村建设全覆盖。加快区域统筹发展。强化分类指导，推动西中东区域互动发展。推进太行山高速、机场三期、聊—邯—长客运专线、城市轨道交通、城市外环路等交通基础设施建设。推动两个文明协调发展。培育和践行社会主义核心价值观，加强历史文化名城保护，打造特色文化品牌，构建现代公共文化服务体系，全面推进文化体制机制创新，加快建设文化强市，力争建成全国文明城市。

（四）坚持走绿色发展之路，致力打造生态宜居美丽家园。把绿色作为加快发展的重要保障，牢固树立绿水青山就是金山银山的理念，坚定不移走绿色循环低碳发展之路。全面改善大气质量。实施主城区污染排放大户搬迁改造工程，深化燃煤污染、工业污染、扬尘污染和机动车尾气治理，全市主要污染物排放总量持续下降，力争退出全国空气质量排名“后十位”。积极创建国家森林城市。开展大规模国土绿化行动，实施太行山绿化和“矿山披绿”三年攻坚行动，每年造林绿化52万亩以上，森林覆盖率达到35%左右，让绿色成为邯郸的永久底色。强化生态修复保护。加强矿山治理、河流整治及重点生态功能区恢复，加快地下水超采综合治理，搞好饮用水源地保护，加大土壤和农业面源污染防治力度，优化生态系统，强化生态支撑。节约高效利用资源。坚持严格的耕地保护和集约节约利用土地制度，实行能源和水资源消耗、建设用地等总量和强度双控制，大力发展循环经济，推广使用清洁能源，推动形成绿色发展方式和生活方式。改善生态环境是最大的民生，我们一定驰而不息、奋力攻坚，努力让邯郸的天更蓝、水更清、地更绿、空气更清新！

（五）坚持走开放发展之路，积极构建合作共赢崭新格局。把开放作为加快发展的必由之路，以更高水平的开放促进更高质量的发展。打造开放新平台。发挥经济技术开发区、冀南新区引领作用，提升各县（市、区）产业园区建设水平，建设综合保税区、国别（地区）产业园等开放合作新平台，聚集优质项目。拓展开放新空间。更加积极主动融入京津冀协同发展，最大限度用好京津优质资源，打造京津先进制造业转移承接地、食品工业扩能首选地、优质医疗教育资源合作优选地和旅游目的地。广泛参与中原经济区分工协作，深化与环渤海地区的产业合作，全方位扩大对内对外开放。提升开放新层次。积极承接京津、长三角、珠三角、长江经济带产业转移，引进一批战略投资者。支持企业主动参与国际竞争，促进对外贸易优化升级，推进跨境电子商务发展，全面提高开放型经济发展水平。

（六）坚持走共享发展之路，努力让改革发展成果普惠于民。把共享作为加快发展的根本目的，“十三五”期间继续加大民生投入，让人民群众在共建共享中有更多获得感。实施脱贫攻坚工程。坚持精准扶贫、精准脱贫，确保到2018年贫困县全部摘帽，贫困村全部出列，贫困人口全部脱贫。实施就业创业扶持工程。完善就业创业服务体系，新增城镇就业50万人、农村富余劳动力转移就业20万人，城镇失业人员再就业15万人。实施社保扩面提标工程。建立统一的城乡居民基本医疗保险制度，全面完善社会保障体系，扩大社保覆盖面，健全住房保障和供应体系，提高保障能力和水平。实施教育提质惠民工程。均衡配置城乡教育资源，高水平普及学前三年教育，推进义务教育均衡发展，全面提高普通高中教育质量，支持现代职业教育和特殊教育发展，提升高等教育办学水平，完善家庭困难学生资助体系，促进教育公平。实施“健康邯郸”工程。系统推进医疗、卫生、养老、康复等大健康产业发展和服务体系建设，打造区域医疗中心。实施食品药品安全工程。建立全程可追溯制度，提升安全监管水平，确保全市人民“舌尖上的安全”。实施公共安全工程。深化平安邯郸建设，加强和创新社会治理，有效预防和化解矛盾纠纷，维护社会和谐稳定大局。

各位代表！把邯郸的明天建设得更加美好，是全市人民的共同期盼。只要我们在市委的坚强领导下，紧紧依靠全市人民，解放思想、抢抓机遇、奋发作为，全面建成小康社会的目标就一定能够实现，一个更加富强、更加美丽、更加幸福的新邯郸必将呈现在世人面前！

三、2016年主要工作

2016年是“十三五”开局之年，也是推进供给侧结构性改革的攻坚之年，更是为全面建成小康社会奠定坚实基础的关键之年，做好今年的工作意义重大。

综合考虑各方面因素，全市经济社会发展的主要预期目标是：全市生产总值增长7%，一般公共预算收入增长7%，规模以上工业增加值增长5.5%，固定资产投资增长10%左右，社会消费品零售总额增长10%左右，出口总值和实际利用外资均增长5%。城镇、农村居民人均可支配收入分别增长8%和8%以上，居民消费价格涨幅控制在3%左右，城镇登记失业率控制在4.5%以内。PM2.5平均浓度下降率、单位生产总值能耗和化学需氧量、氨氮、二氧化硫、氮氧化物等主要污染物排放量完成省定目标。

实现上述目标，重点把握好四个方面：一是必须坚持“稳中快进”的总基调，进一步增强看齐意识和压力意识，扭住发展不放松，强势开局，逆势而上，跨越赶超；二是必须准确把握供给侧结构性改革要求，全力抓好去产能、去库存、去杠杆、降成本、补短板任务，实现低水平供需平衡向高水平供需平衡跃升；三是必须把转方式调结构摆在更加突出位置，把稳增长和促转型、优增量和调存量有机结合起来，努力追求更高质量和效益的发展；四是必须把问题导向贯穿始终，勇于改革、大胆创新、破解难题，推动经济又好又快发展。

重点做好十个方面工作：

（一）突出有效投资，构筑持续发展新支撑。进一步优化投资结构，提高投资效益，以优质增量扩总量、稳增长。强化重大项目带动。加快推进武安新峰煤制天然气、峰峰鑫宝新材料等100个开工在建项目，大名子丰多肽产业园、广平极度电动车等50个竣工投产项目，成安星星白色家电等50个重大前期项目，形成良好的梯次建设格局，努力实现投资有回报、产品有市场、企业有利润、员工有收入、政府有税收、环境有改善。强化产业园区支撑。继续加大园区投入，完善配套设施，提高园区聚集项目水平。探索实施引进项目“飞地”政策，实行税收共享，引导项目集群发展。充分发挥经济技术开发区、冀南新区引领带动作用，省级以上开发区财政收入、规模以上工业增加值等主要指标实现大幅增长，把开发区打造成对外开放的主战场、经济发展的“火车头”。支持各县（市、区）打造特色产业园区，抓好馆陶铠特农机、魏县台资装备制造等“园中园”建设，搭建一流平台，聚集优质产业。强化各类要素保障。严格落实重点项目“亩产效益综合评价体系”，倒逼企业高效利用土地。对闲置两年以上的土地依法收回，对长期不能投产达效的企业通过重组或破产，实现“腾笼换鸟”，最大限度盘活存量用地。成立工业投资公司，设立战略性新兴产业扶持基金和中小企业发展基金，推介发布PPP重大项目，吸引社会资本广泛参与。强化重大基础设施建设。启动107和309国道市区段改建工程，争取城市轨道交通建设规划早日获批，东郊热电14号机组竣工投用。

（二）调整产业结构，增创转型突破新优势。坚决闯过结构调整这一历史关口，推动产业提质增效升级。全年技改投资增长12%以上，高新技术产业增加值增长10%以上。挖掘传统产业增长潜力。制定传统产业改造提升计划，强力推进钢铁产业减量置换、整合重组，加快太行、冀南钢铁退城进园升级改造和宝信钢铁转型升级项目进度，永洋特钢退城搬迁升级改造项目一期工程竣工投产。引导煤炭行业转型突破，支持峰峰集团、邯矿集团延长产业链条，实现多元发展。坚决化解过剩产能。把化解过剩产能任务落实到县（市、区）、企业和装备，全面完成省定任务。抓住处置“僵尸企业”这个牛鼻子，按照企业主体、政府推动、市场引导、依法处置的办法，通过兼并重组、寻求战略合作，力争实现市场出清。培育壮大战略性新兴产业。重点发展先进装备制造、节能环保、新能源汽车、新材料、电子信息等产业，支持经济技术开发区捷克产业园、肥乡云计算产业园、邯郸县凯盛科技园等做大做强，壮大武安银隆新能源汽车和电池材料项目规模，力争三年产值超千亿元。强化实体经济支撑。深入开展“企业服务年”活动，积极帮助企业降低交易、人工、财务等成本。筛选100家传统产业转型升级企业、100家战略性新兴产业企业、100家科技型中小企业，进行重点帮扶。提高产需对接实效，鼓励引导市内各单位、各企业优先采用本地产品，相互支持，抱团取暖。加强质量标准品牌建设，增强企业竞争力。积极推进“个转企、小升规”，确保新增入统工业企业150家以上。

（三）加快拓展三产，培育现代服务业新亮点。重点实施“345”工程，抓好30个现代物流项目、40个文化旅游项目、50个其它生产性服务业项目，推动现代服务业发展提速、比重提高、结构提升，服务业增加值增长10%以上。提升商贸物流业态层次。发挥冀中能源国际物流、万合集团等龙头企业带动作用，大力发展第三方和第四方物流，加快华耀城、林安智慧物流、春生农业物流、传化公路港物流等项目建设，打造主城区南部商贸物流聚集区。启动青岛保税港区邯郸（鸡泽）功能区建设，确保武安保税物流中心封关运行。积极发展电子商务，抓好阿里巴巴农村淘宝、河北网商园二期等电商项目建设。积极谋划引进区域性快递分拣中心项目，促进快递业加快发展。做大做强文化旅游业。推进广府古城争创国家5A级景区，加快华强中华成语文化博览园、东太行景区、北响堂山景区升级改造等文化旅游项目建设。支持文化旅游产业集团发展，加强与中景信等旅游公司的战略合作，提升娲皇宫等景区开发管理水平。加大区域景区整合和太行山生态保护力度，结合传统村落保护和美丽乡村建设，打造高水平乡村旅游片区。加强旅游协作，推动联合发行“金中原旅游一卡通”，开发文化旅游纪念品，有效促进旅游消费。强力推进旅游路网建设，打造精品线路，接待国内外游客人数、旅游总收入分别增长10%和20%以上。繁荣活跃金融业。深入开展“金融创新年”“企业上市年”活动，加快培育区域性资本市场，壮大市金融资本服务中心和金融发展中心，年内入驻金融机构20家以上。支持邯郸银行在京津开展业务，争取引进3家银行、3家保险公司，新建3支以上投资基金。加大企业上市力度，新增挂牌上市企业60家以上。创优金融生态环境，防范金融风险，建立企业信用信息平台，依法打击恶意逃废银行债务行为，深入落实“金十条”“抵质押九条”，引导金融机构千方百计扩大信贷投放，支持实体经济持续健康发展。有力促进消费需求。扩大信息、绿色、住房、旅游休闲、教育文体、养老健康家政等六大领域消费。加快互联网宽带示范城市和高速宽带网络建设，健全农村市场流通体系，释放农村消费潜力。完善落实“房九条”，打通保障房和商品房等供需通道，推行棚户区改造货币化安置，发展住房租赁市场，推进以满足新市民住房需求为出发点的住房制度改革，提高住房公积金使用率，有效化解房地产库存，促进房地产业平稳健康发展。

（四）夯实“三农”基础，开创农业农村新局面。加快转变农业发展方式，让农业更强、农民更富、农村更美。大力发展现代农业。落实藏粮于地、藏粮于技战略，加强农业基础设施建设，提高农业综合生产能力。以园区建设为载体，以产业化经营为主线，重点实施“1515”工程，建成15个市级以上现代农业园区，推进100个农产品加工项目，完成年度投资50亿元以上。促进一二三产业融合发展，每个县（市、区）至少打造2个示范点。培育壮大重点龙头企业，突出发展粮油、果蔬、肉类、乳制品等精深加工，提高农产品加工增值率，提升农产品供给质量，农业产业化经营率达到66.8%以上。加快建设美丽乡村。按照“环境美、产业美、精神美、生态美”的要求，在净、绿、富的基础上，实施民居改造、污水治理、街道硬化、饮水安全巩固提升等专项行动，集中打造一批整体推进县和连片示范区，完成500个重点村建设任务。深化农村改革发展。农村土地确权颁证面积达到90.8%，建设4个县级农村产权流转交易平台，培育新型职业农民，壮大农村经营主体，依法推进土地经营权有序流转，让农民资产活起来、农村土地要素活起来。全力打好脱贫攻坚战。持续加大扶贫投入，建设精准扶贫信息平台，把扶贫开发与培育富民产业、完善基础设施、建设美丽乡村、发展乡村旅游紧密结合起来，实行农村低保线与扶贫线“两线合一”，强化贫困群众技能培训，引导贫困群众自力更生、艰苦创业，靠自己的勤劳和智慧建设美好家园，确保16.85万贫困人口稳定脱贫。

（五）推进创新创业，打造经济增长新引擎。更加注重创新供给，让创新在全社会蔚然成风。加快协同创新。用足用好京津创新资源，积极引进京津科技成果、领军人物、高端人才和创新团队。搭建协同创新平台，加快北大邯郸创新研究院高科技项目产业化，建设国家领军人才工作站、中国青年海归工作站、北京科学技术研究院邯郸分院，促进京津科技创新要素在我市聚集。强化自主创新。围绕先进装备制造、节能环保等新兴产业，实施一批重大科技项目，提高自主创新能力。加快冀南新区国家级现代装备制造创新型产业集群建设。全面落实“科技九条”，充分发挥科技创业投资和成果转化引导基金作用，新增科技型中小企业1000家以上、高新技术企业20家以上。激发“双创”活力。积极落实各级支持创新创业政策措施，鼓励发展众创、众包、众扶、众筹等新模式，加快各类创业支撑平台建设，各县（市、区）至少建设1家科技孵化器和1家众创空间，最大限度地激发大众创业、万众创新热情。

（六）深化改革开放，拓展经济发展新空间。以改革统领全局，以开放活跃全局，不断为经济发展注入新动力。深化重点领域改革。加快“放管服”改革，大力推行网上审批，规范升级“三级平台、两个代办”，清理规范行政许可中介服务事项，建立统一规范的公共资源交易平台。启动园区管理体制改革。深化国有企业改革，完成8家破产企业职工安置扫尾工作。推进供销社改革。加快城市公立医院改革，实现人民群众得实惠、医务人员受鼓舞、医院发展可持续。深化财税体制、政府采购体制改革，在城市管理上积极推行政府购买服务。完成市县公车改革。全方位扩大开放。围绕“215”目标，进一步加大招商引资力度。主动融入京津冀协同发展，精心组织开展“走进百家央企”活动，市级4个招商部、经济技术开发区和冀南新区分别对接央企10家以上，各县（市、区）分别对接5家以上，持续引进一批重大合作项目。落实出口退税政策，扩大外贸出口，提高机电装备和高新技术产品出口规模。实施企业“走出去”战略，开展国际优势产能和先进装备合作，提高经济外向度。大力优化发展环境。加大明查暗访力度，集中整顿不作为、慢作为、乱作为等突出问题和企业周边环境，对吃拿卡要等典型案件坚决查处并公开曝光。全力支持民营企业开展“二次创业”，努力激活民间资本，增强民营经济发展活力。

（七）科学精准治污，力促环境质量新提升。以壮士断腕的勇气，坚决打好环境治理攻坚战。深入推进大气污染防治。实施500项减排治理工程，削减煤炭消费量130万吨。大力开展焦化行业、道路车辆、露天矿山和散煤污染整治四大专项行动，突出治理车、油、路污染，加快充电设施建设，积极推广使用新能源汽车，实行载重货车绕行主城区。实施工业企业污染减排、集中供热、挥发性有机物治理等六大工程，对主城区建筑工地扬尘治理全部在线监测，实施主城区25.5万户焦炉煤气置换天然气工程，在县城大力推行“煤改气”，加快推进主城区4家污染排放大户搬迁改造工程。高度重视水环境改善。认真落实最严格的水资源管理制度和水污染防治行动计划，强力推进东区防洪排涝体系工程建设，对滏阳河、洺河进行全流域退污还清治理，加快马头分洪道、永年娄里水库和涉县茅岭底水库前期立项审批。实施“清水入沁”，完成南水北调配套工程扫尾，抓好地下水超采综合治理和引黄入冀补淀邯郸段工程。加强岳城水库、羊角铺等饮用水源地保护，治理水土流失面积150平方公里，积极争列全国合同节水试点城市。持续推进“绿美邯郸”建设。集中打造太行山绿化、紫山绿化、廊道绿化、村庄绿化等重点工程，完成造林绿化54万亩，森林覆盖率新增1.5个百分点。严格环保监管执法。充分发挥市空气质量保障应急指挥中心作用，统一指挥，联防联控，强化监测，提高治理的针对性。严格落实《环境空气质量生态补偿办法》，进一步强化县级治污责任，实现主城区和各县（市、区）同步治理。加大环境执法力度，对偷排偷放和超标排放等违法行为，依法查处，严惩不贷。

（八）坚持以人为核心，推动新型城镇化取得新成效。统筹推进城市规划建设、经济发展、管理服务，进一步强化邯郸在京津冀世界级城市群中区域性中心城市地位。抓规划，优化城市布局。强化规划引领，启动新一轮城市总体规划修编，完善专项规划体系，推动“多规合一”，构建中心城市、县城、小城镇良性发展的城镇化格局。抓建设，完善承载功能。把东区开发作为城市建设的重中之重，加快推进河北工程大学迁建、市第一医院新区等重点工程建设，确保客运枢纽一期、科技中心、青少年活动中心竣工投用。高标准建设中央商务区，启动地下环路、综合管廊建设，加快已启动城中村征迁改造、回迁房和园林水系、路网管网等配套工程建设，打造现代化宜居新城。按照便民、急需的原则，搞好老城区提档升级。同时，大力推广绿色建筑，加快住宅产业化基地建设。抓提升，繁荣城市经济。坚持以产兴城、以城带产、产城融合，在老城改造和新城建设中优化城市产业布局，突出发展高端服务、现代商贸、信息中介、创意创新、高新技术等新兴产业，提高经济容积率。抓管理，打造洁净城市。实施城市“洁净工程”，对市区西环路、南环路及老旧小区、背街小巷、城乡结合部等重要路段和节点进行环境综合整治，彻底治理垃圾乱倒、摊点乱摆、广告乱贴等突出问题。实行各级领导精细化管理现场巡查制，从市、区县和城管部门主要负责同志做起，带头徒步走街串巷，到一线发现问题、解决问题，尽快让城市干净起来、整洁起来。抓配套，加快农业转移人口市民化。积极落实户籍制度改革政策，促进居住证制度落地，推动城镇常住人口基本公共服务均等化，常住人口城镇化率提高2个百分点。

（九）壮大县域经济，实现增比进位新突破。把县域经济作为“稳中快进”的重要支撑，进一步做大规模，做强产业，做出特色。做大县域经济总量。积极开展对标行动，制定跨越赶超计划，力争更多的县进入全省三十强。支持西部县（市、区）加速转型升级，实现新旧动能转换。支持东部县加快振兴崛起，进一步扩总量、上规模、增实力，主要经济指标增速实现两位数增长。支持中部县（区）聚集先进要素，突出快中更优，实现更高质量的发展。参照省考核办法，实行县域经济发展排名月通报制度，调动发展县域经济积极性。做强特色产业集群。制定出台支持精品钢材、先进装备制造、食品加工等特色产业集群发展的意见，一个产业集群一个推进计划。大力发展园区经济，支持乡域经济发展。每个县（市、区）着力打造1—2个主业突出、特色鲜明的产业集群，推动曲周自行车、永年紧固件、成安制管、邱县食品、鸡泽铸造等扩规模、上档次、增效益，提升特色产业集群发展水平。启动县城建设三年攻坚行动。坚持走“小县大县城”的路子，加快推动产城教融合发展，支持有条件的县（市、区）规划建设一个功能完善的教育园区。提升县城建设水平，突出特色、传承文化、注重生态、提升品位。着力完善服务功能，年内所有县城全部实现集中供水、供暖，污水、垃圾达标处理，增强县城聚集人口和产业能力。

（十）保障改善民生，满足人民生活新期待。越是经济困难，越要更加注重保障和改善民生。办好一批民生实事。重点实施8个方面76件民生实事。新增城镇就业10万人，下岗失业人员再就业3万人。完善社会保障体系，进一步扩大社保覆盖面。推行医养结合，新建30个农村精品幸福院、25个社区居家养老服务中心。建立购租并举的住房制度，扩大住房保障范围，开工建设或购买保障性住房、棚户区改造住房14803套。全面发展社会事业。实施第二期学前教育三年行动计划，统一城乡义务教育经费保障机制，建成50所农村标准化中小学，改善薄弱学校和寄宿制学校办学条件，加快主城区西部省级示范性高中建设，提高教育教学质量。提升医疗服务水平，创建“健康家庭”，推进分级诊疗，落实全面两孩政策。加快构建现代公共文化服务体系，加强文物保护，争取邺城遗址列入国家考古遗址公园立项名单。广泛开展全民健身运动，举办好第十三届国际太极拳运动大会。积极创新社会治理。加强对各种风险源的研判分析，有效化解风险隐患。严格落实安全生产责任制，坚持关口前移，更加注重预防，加强隐患排查整改和高危行业领域专项整治，坚决遏制重特大生产安全事故发生。加大食品药品安全监管力度，确保全市人民饮食用药安全。高度重视和加强信访稳定工作，建立社会矛盾多元化解机制。持续做好防范和处置非法集资工作，完善立体化社会治安防控体系，为人民群众创造安定和谐的社会环境。支持国防和军队建设改革，加强国防后备力量建设，统筹做好新闻出版广电、气象防震减灾、民族宗教、邮政通信、人民防空、妇女儿童、扶残助残、老龄等各项工作。

四、全面加强政府自身建设

实现“十三五”目标、全面建成小康社会，责任重于泰山，使命无上光荣。我们一定恪尽职守、夙夜在公、为民服务，奋力开创政府各项工作新局面。

（一）深入解放思想，提高开拓创新能力。把创新贯穿政府工作全过程，打破惯性思维，破除路径依赖，靠改革破难前行，用创新突破瓶颈。坚持高标定位、事争一流，勇于在破除旧框框中探求新方法，在继承发展中谋划新思路，在借鉴先进经验中形成新举措，确保政府的每一项决策部署都能体现时代性、把握规律性、富有创造性。

（二）加快转变职能，提高服务发展能力。深化行政审批制度改革，坚决取消一切束缚经济社会发展的行政权力，精简办事程序，缩短办理时限，做到目录之外无审批、中心之外无循环，最大限度方便企业、方便群众、方便基层。坚决向不作为和懒政怠政行为“亮剑”，不断激发政府工作人员干事创业的热情和活力。

（三）加强法治建设，提高依法行政能力。坚持依法科学民主决策，严格按照法定权限和程序行使权力、履行职责，提高运用法治思维和法治方式化解矛盾、推动发展的能力。自觉接受市人大法律监督和工作监督、市政协民主监督，高度重视社会和新闻舆论监督，保障人民群众的知情权、参与权、表达权和监督权。

（四）持续改进作风，提高履职尽责能力。崇尚实干、力戒空谈。深入开展机关作风整顿，始终保持奋发有为、昂扬向上的精神状态，把全部心思和精力聚焦到加快发展上，夙兴夜寐，激情工作，勇于担当，对形成的决策、议定的事项，言出行随，说了就算，定了就干，干就干成，以发展的实际成效取信于民。

（五）时刻勤勉自律，提高廉洁从政能力。始终把纪律和规矩挺在前面，严格落实中央“八项规定”精神，认真践行“三严三实”，自觉遵守《准则》和《条例》，落实“两个责任”，强化“一岗双责”，加强工程项目、土地出让、公共资源等重点领域监管，严厉查处损害群众利益的腐败行为，树立为民务实清廉的良好形象。

各位代表，蓝图已绘就，扬帆正当时。让我们在省委、省政府和中共邯郸市委的坚强领导下，以更大的勇气改革创新，以更强的毅力攻坚克难，以更实的作风干事创业，为加快建设富强邯郸美丽邯郸、全面建成小康社会而努力奋斗！
